# Neurocognition after prenatal levetiracetam, lamotrigine, carbamazepine or valproate exposure

**DOI:** 10.1007/s00415-020-09764-w

**Published:** 2020-02-28

**Authors:** Yfke Huber-Mollema, Loretta van Iterson, Frans J. Oort, Dick Lindhout, Roos Rodenburg

**Affiliations:** 1 Stichting Epilepsie Instellingen Nederland (SEIN), Heemstede, The Netherlands; 2 Research Institute of Child Development and Education, University of Amsterdam, 15776, 1001 NG Amsterdam, The Netherlands; 3 Department of Genetics, University Medical Center Utrecht, Utrecht, The Netherlands

**Keywords:** Epilepsy, Pregnancy, Antiepileptic drugs, Cognition, Neuropsychological assessment, EURAP & development

## Abstract

**Objective:**

To examine neurocognitive functioning of children exposed prenatally to carbamazepine, lamotrigine, levetiracetam or valproate monotherapy.

**Methods:**

In a prospective observational study, children aged 6 or 7 years, identified from the European Registry of Antiepileptic Drugs and Pregnancy database in The Netherlands, were assessed using the Wechsler Intelligence Scale for Children and the developmental neuropsychological assessment. Maternal IQ was measured using Wechsler Adult Intelligence Scale. Assessors were blinded to drug exposures.

**Results:**

One hundred and sixty-one children (one set of twins and 21 sibling pairs) of 139 mothers were included. As a group, children achieved average scores on neurocognitive outcomes. Children exposed to valproate (*n* = 22) performed lower on all six neurocognitive domains, especially language, than those exposed to carbamazepine (*n* = 32), lamotrigine (*n* = 82) or levetiracetam (*n* = 25). After controlling for maternal IQ and drug dose, the verbal IQ of valproate-exposed children was on average 9.1 points lower than those exposed to carbamazepine (95% confidence interval [CI] 1.3–17.0; *p* = 0.023), 10.3 lower than lamotrigine-exposed children (CI 3.4–17.3; *p* = 0.004) and 13.4 lower than levetiracetam-exposed children (CI 5.2–21.6; *p* = 0.002). No significant dose–effect was found. Virtually no significant differences were found between lamotrigine and levetiracetam or lamotrigine and carbamazepine exposed children.

**Conclusions:**

Consistent with previous research, valproate-exposed children experienced more problems compared to three other common antiepileptic drugs, while children exposed to lamotrigine, carbamazepine or levetiracetam revealed little to no problems. This illustrates the need for systematic follow-up of prenatally exposed children, to support pre-pregnancy counseling and treatment decisions in women of reproductive age.

**Electronic supplementary material:**

The online version of this article (10.1007/s00415-020-09764-w) contains supplementary material, which is available to authorized users.

## Introduction

Epilepsy affects up to 1% of the population [1], and antiepileptic drugs (AEDs) are the main treatment. About a third of people receiving AEDs are women of reproductive age [2], and women in three to four per 1000 pregnancies take AEDs [3]. Treatment continuation during pregnancy is a must for most women with active epilepsy [4].

Knowledge of AED teratogenicity has increased in the past decade [5, 6]. Children of mothers with epilepsy are at higher risk of congenital malformations and, for a number of maternal AEDs, these risks show a dose–effect relationship [7]. Increasing attention has also been paid to the long-term neurocognitive and behavioral effects of prenatal exposure to AEDs [6]. The greatest impact has been observed for valproate (VPA) [5, 6, 8, 9]. As this drug is associated with major malformations and neurocognitive effects it is no longer routinely prescribed to women with child-bearing potential [10], which has resulted in increased use of newer AEDs such as lamotrigine (LTG) and levetiracetam (LEV) [11, 12]. To date, few or no effects on neurocognition have been found with carbamazepine (CBZ) or LTG [6, 13]. However, currently available data on long-term development of prenatal LTG exposed children are fairly limited, and even less is known about possible effects of LEV [8, 14].

We examined neurocognitive functioning of children aged 6 or 7 years, prenatally exposed to monotherapy with CBZ, LTG, LEV or VPA. It was hypothesized that children exposed to VPA would have impaired neurocognitive functioning compared to children exposed to CBZ, LTG or LEV. In addition, LTG and LEV were exploratively examined as these are first choice treatments for many women with epilepsy of childbearing age.

## Methods

### Study design and participants

We collaborated with the European Registry of Antiepileptic Drugs and Pregnancy (EURAP) to design the Dutch EURAP & Development study [15]. EURAP & Development is a prospective observational study of children of mothers with epilepsy, with assessors blinded to drug exposures. The current study is part of a larger longitudinal study in which long-term effects of prenatal exposure to AEDs on neurocognitive and behavioral development are investigated from a family perspective [15].

Participants were mother–child pairs identified from the EURAP-NL database in The Netherlands, a national, single center pregnancy register that investigates the prevalence of major congenital malformations following prenatal exposure to AEDs. Women are enrolled by the EURAP-NL center through self-referral or by their health professional. Recruitment occurs preferably within the first 16 weeks of pregnancy—relevant for the evaluation of major malformations—facilitating prospective information about health and well-being during the pregnancy [7]. Mother–child pairs with risk factors (e.g., seizure occurrence, alcohol or nicotine use during pregnancy) assessed prenatally, after delivery, or up until 3 years of age, were eligible. Inclusion criteria were maternal CBZ, LTG, LEV or VPA monotherapy starting before conception and continuing during the entire pregnancy, and the child aged between 6.0 and 7.11 years at the neurocognitive assessment. Children were excluded if (1) the mother was unable to take care of the child (e.g., lives in foster care), (2) the child has a known chromosomal/genetic syndrome or prematurity (gestational age less than 37 weeks), or (3) there were factors other than AED exposure which significantly modified child development, such that reliable assessment was not possible.

As participants lived all across The Netherlands, the study was conducted at different locations [e.g., Heemstede (epilepsy center SEIN), Amsterdam (University of Amsterdam), Rotterdam, Zwolle and Groningen (outpatient clinics SEIN), Nijmegen (Radboud University), Eindhoven (center for child psychiatry) and Heeze (epilepsy center Kempenhaeghe)]. If travel to one of the study locations was not possible, the child assessment took place at home. All assessors (in total thirteen child psychologists, including (and under supervision of) YH-M) were (video) trained and monitored according to the test protocol, to ensure standardized procedures. Further detailed information on procedures are provided in the study protocol [15].

### Measures

#### General information

Parents completed an online questionnaire on demographic information, developmental milestones, school performance and additional educational needs.

#### Intelligence

Nine subtests of the Wechsler Intelligence Scale for Children (WISC-III-NL) [16] were used to assess child intelligence: picture completion; information; object assembly; similarities; block design; comprehension; coding; symbol search and digit span. The WISC-III short form assesses full-scale IQ (FSIQ), verbal IQ (VIQ), performance IQ (PIQ) and the processing speed index (PSI) [17]; parents completed the short form of the Wechsler Adult Intelligence Scale (WAIS-III-NL) [18] (seven subtests).

#### Attention and executive functioning

We used the subtests auditory attention, response set, inhibition, statue, and design fluency of the developmental neuropsychological assessment (NEPSY-II-NL) [19], which allows the measurement of subcomponents of attention and executive functions. These are: inhibition of learned and automated responses; monitoring and self-regulation; alertness, selective and sustained attention; ability to establish maintain and change responses; nonverbal problem solving, planning and organizing a complex response; and production of patterns [19]. In contrast to the first edition of the NEPSY, NEPSY-II-NL does not include a visual attention task. We therefore used the Visual Sky Search task of the Test of Everyday Attention for Children (Tea-CH) [20]. With this task the child is asked to search an A3 sheet with numerous pairs of spaceships which are randomly distributed and to try to circle as many pairs of identical spaceships as quickly as possible.

#### Language skills

From the language domain of the NEPSY-II-NL, we assessed speeded naming, comprehension of instructions, and word generation. These subtests measure fast semantic access to, and production of, words (e.g. names of colors, shapes, or sizes); the ability to receive, process, and execute oral instructions of increasing complexity; and verbal productivity through the ability to generate words within specific semantic categories [19]. We used the Peabody Picture Vocabulary Test (PPVT-III-NL) to measure vocabulary [21]. Verbal fluency was assessed with the Lindeboom [22]. This is a short confrontational naming task where the child is asked to name rapidly 15 common pictures. The time score is used as outcome measure, with shorter times indicating better performance. To measure phonological processing (not included in the NEPSY-II-NL), we applied two short language tasks: auditory synthesis (sound blending) [23] and phoneme deletion [24].

#### Memory and learning

Short and long-term memory were measured with memory for faces, memory for faces delayed, memory for names, memory for names delayed and narrative memory of the NEPSY-II-NL. The subcomponents that were assessed included: encoding of facial features, as well as face discrimination and recognition; the ability to learn names of children; and the ability to remember organized verbal material, under free recall, cued recall and recognition conditions [19].

#### Fine motor skills

From the Sensorimotor domain of the NEPSY-II-NL fingertip tapping, imitating hand positions, and visuomotor precision were measured for fine motor skills. The subcomponents that were assessed included the ability to imitate hand positions, to produce repetitive and sequential finger movements and to use a pencil with speed and precision [19]. Handedness was observed during the assessment.

#### Visuospatial skills

From the visuospatial processing domain of the NEPSY-II-NL, arrows and design copying were used to measure visuospatial skills. The subcomponents that were assessed included the ability to judge line orientation and the ability to copy two-dimensional geometric figures with paper and pencil [19].

### Statistical analyses

Data were analyzed using IBM SPSS Statistics 24. Descriptive analyses were performed for each AED taken, to describe the sample and to examine the nature and severity of neurocognitive development and the frequency of additional educational needs. All neurocognitive measures are standardized by age of the child based on population norms from the different test manuals. Some raw scores of the NEPSY-II-NL are originally standardized as percentile scores [19]. To facilitate interpretation, all NEPSY-II-NL scores were transformed to standard scores with mean 10 and standard deviation 3. Scores between 8 and 12 are considered average; scores of 7 or lower are interpreted as below average and scores of 12 or higher as above average. IQ scores (WISC-III-NL; PPVT-III-NL) have an average of 100, with scores lower than 90 interpreted clinically as below average and scores of 110 and higher classified as above average [25]. In line with DSM classifications, the statistical cut-off score of < 85 is used for percentage of children with below average intelligence [26].

We performed multiple regression analyses for each neurocognitive outcome to test the hypothesis that VPA-exposed children have impaired neurocognitive functioning compared to those exposed to the other AEDs. As LTG was the largest group, and as we also wanted to make a comparison between LTG- and LEV-exposed children (as first choice treatments for many women with epilepsy of childbearing age) and between LTG- and CBZ-exposed children, we performed additional analyses with LTG as reference group. As our sample also included a number of siblings, we conducted multilevel regression analyses to account for within family dependencies.

Potential confounders were selected by assessing their relationships with the medication and outcome variables (through ANOVA with post hoc Tukey tests, Kruskal–Wallis, Chi square, Fisher’s exact tests and Pearson correlations). Variables included as potential confounders were: type of maternal epilepsy; tonic–clonic seizures during pregnancy; use of folic acid; alcohol and nicotine exposure during each trimester; breastfeeding; maternal age at delivery; maternal IQ and educational level; gestational age; gender; age at assessment; presence or absence of congenital malformations and time of inclusion in the EURAP-NL database (Table 1). Variables showing a relationship (*p* < 0.15) with medication and outcome measure, or that were expected to influence child development (e.g., maternal IQ) were entered one by one, each into a separate multiple regression analysis. Variables related to AED use were maternal age at delivery, gestational age, age at assessment, epilepsy type, alcohol use during the first trimester, nicotine use during each trimester, and presence of congenital malformations. As these variables were not found to be related to the outcome measure, we included only maternal IQ in the multiple regression analyses (see bivariate correlations between potential confounders and cognitive outcome measures in the supplemental material, e-Table1a). AED exposure type was entered into the model, with the VPA-exposed group as the reference group. To enable additional comparisons, we repeated the analyses with the LTG-exposed group as group of reference.

**Table 1:** Group demographic information by antiepileptic exposure group

Sample size	VPA	CBZ	LTG	LEV	*p* value
	22	32	82	25	
Maternal characteristics: epilepsy and pregnancy information					
AED daily dose 1st trimester, mg/day mean (range min max)	913.6 (500–1500)	656.3 (200–1400)	277.4 (50–600)	1120.0 (250–3000)	NA
AED daily dose 3rd trimester, mg/day mean (range min max)	940.9 (500–1500)	656.3 (200–1600)	334.2 (50–1000)	1150.0 (250–2500)	
Dose changes, *n* (%), increased/decreased	3 (14%) 2 vs 1	3 (9%) 2 vs 1	50 (61%) 49 vs 1	6 (24%) 4 vs 2	
Maternal age at birth of baby, mean (SD)	33 (3)	32 (5)	31 (4)	32 (4)	.018^a^
Maternal FSIQs^d^, mean (SD)	103 (14)	100 (17)	104 (14)	108 (15)	0.287^a^
VIQs, mean (SD)	102 (13)	100 (16)	102 (13)	105 (13)	0.548^a^
PIQs, mean (SD)	104 (9)	100 (10)	104 (9)	106 (11)	0.125^a^
Maternal education^d^, *n* (%) higher education	12 (60%)	14 (47%)	41 (61%)	14 (70%)	0.720b
Folate supplementation, *n* (%) yes^†^	19 (91%)	23 (77%)	67 (82%)	21 (84%)	0.693^c^
Alcohol exposure, *n* (%) yes					
First trimester	3 (14%)	3 (9%)	26 (32%)	4 (16%)	0.036^c^
Second and/or third trimester	1 (5%)	1 (3%)	8 (10%	0	0.369^c^
Nicotine exposure, *n* (%) yes					
First trimester	6 (27%)	3 (9%)	3 (4%)	1 (4%)	0.007^c^
Second and/or third trimester	3 (14%)	0	2 (2%)	1 (4%)	0.064^c^
Maternal epilepsy type, *n* (%)					0.000^c^
Generalized	16 (73%)	2 (6%)	22 (27%)	9 (36%)	
Localization-related	4 (18%)	28 (88%)	52 (63%)	15 (60%)	
Unknown	2 (9%)	2 (6%)	8 (10%)	1 (4%)	
Tonic–clonic seizures, *n* (%) yes	2 (9%)	5 (16%)	14 (17%)	4 (16%)	0.843^b^
Breastfeeding, *n* (%) yes	5 (23%)	12 (38%)	18 (22%)	4 (16%)	0.261^c^
Paternal characteristics					
Paternal FSIQ^e^, mean (SD)	108 (13)	104 (14)	111 (13)	113 (11)	0.192^a^
VIQs, mean (SD)	103 (16)	104 (12)	110 (13)	113 (16)	0.120^a^
PIQs, mean (SD)	104 (9)	102 (10)	105 (9)	106 (4)	0.429^a^
Paternal education^e^, *n* (%) higher education	11 (55%)	10 (33%)	43 (63%)	13 (62%)	0.178^b^
Child characteristics					
Age at assessment, months, mean (SD)	81.5 (6.2)	81.0 (6.1)	82.7 (7.7)	78.2 (5.6)	0.051^a^
Gestational age, weeks, mean (SD)	40.6 (1.3)	39.9 (1.4)	40.0 (1.1)	40.3 (1.1)	0.089^a^
Child sex, *n* (%) male	11 (50%)	15 (47%)	42 (51%)	15 (60%)	0.804^b^
Congenital malformations, *n* (%) yes	5 (23%)	4 (13%)	4 (5%)	1 (4%)	0.043^c^
Sibling, *n* (%) yes	2 (9%)	1 (3%)	14 (17%)	4 (16%)	
Inclusion moment EURAP-NL, *n* (%)					0.812^b^
Before 16th week pregnancy	14 (64%)	22 (69%)	62 (76%)	19 (76%)	
Between 16th week and birth	6 (27%)	6 (19%)	11 (13%)	3 (12%)	
After birth	2 (9%)	4 (13%)	9 (11%)	3 (12%)	
Parental report of child needs					
Special education, *n* (%)	2 (9%)	0 (0%)	3 (4%)	0 (0%)	0.227^c^
Repeating a year of school, *n* (%)	4 (18%)	3 (9%)	9 (11%)	1 (4%)	0.491^c^
Additional educational needs, *n* (%)	8 (36%)	5 (16%)	11 (13%)	3 (12%)	0.099^c^
Developmental delay, *n* (%)	7 (32%)	4 (13%)	8 (10%)	3 (12%)	0.088^c^
Physiotherapy, *n* (%)	10 (46%)	9 (28%)	21 (26%)	7 (28%)	0.347^c^
Speech therapy, *n* (%)	7 (32%)	11(34%)	24 (29%)	4 (16%)	0.447^c^

*AED* antiepileptic drug, *VPA* valproate, *CBZ* carbamazepine, *LTG* lamotrigine, *LEV* levetiracetam, *TIQs* estimated total intelligence, *VIQs* estimated verbal intelligence, *PIQs* estimated performance intelligence

^a^Analysis of variance (continuous data)

^b^Chi square

^c^Fisher exact (dichotomous data)

^d^One hundred and thirty-nine mothers. One mother without IQ scores

^e^One hundred and thirty-nine fathers of which 85 with IQ scores

^†^Appropriate use of folic acid was defined as at least 4 weeks before conception with a minimum dose of 0.4 mg/day. Three missing because of unknown start date of folic acid

Dose effect was included in the regression analyses as the percentage relative to the median group AED dose [100 × ((dose first trimester − median group AED dose)/median group AED dose)]. An interaction term between AED type and dose (e.g., VPA dose) was also included separately in the regression models. Correlation analyses were used to examine relationships between AED dose (CBZ, LTG, LEV, VPA) and outcome measures. We examined relationships with first trimester as well as third trimester dose.

Analyses were conducted with all available scores on the outcome variables, without imputation for missing data on outcome variables. Specific neurocognitive scores were missing on two children in the VPA-exposed group, since we only obtained information from their parents. Both children had previously been assessed within a clinical setting; we therefore included IQ scores based on the psychological report and parent information from the online questionnaire. For one mother without IQ scores, we used the average IQ in her education group.

We performed sensitivity analyses with only one child from each family (the first-born child within the study) and with only the children included in EURAP-NL before 16 weeks of gestation to avoid possible bias.

#### Data availability

The study protocol is available on PsyArXiv, https://doi.org/10.17605/OSF.IO/B8DYJ. Anonymized data will be restrictedly available after project completion from the corresponding author on reasonable request by a qualified investigator.

## Results

### Participants

**Fig. 1:**
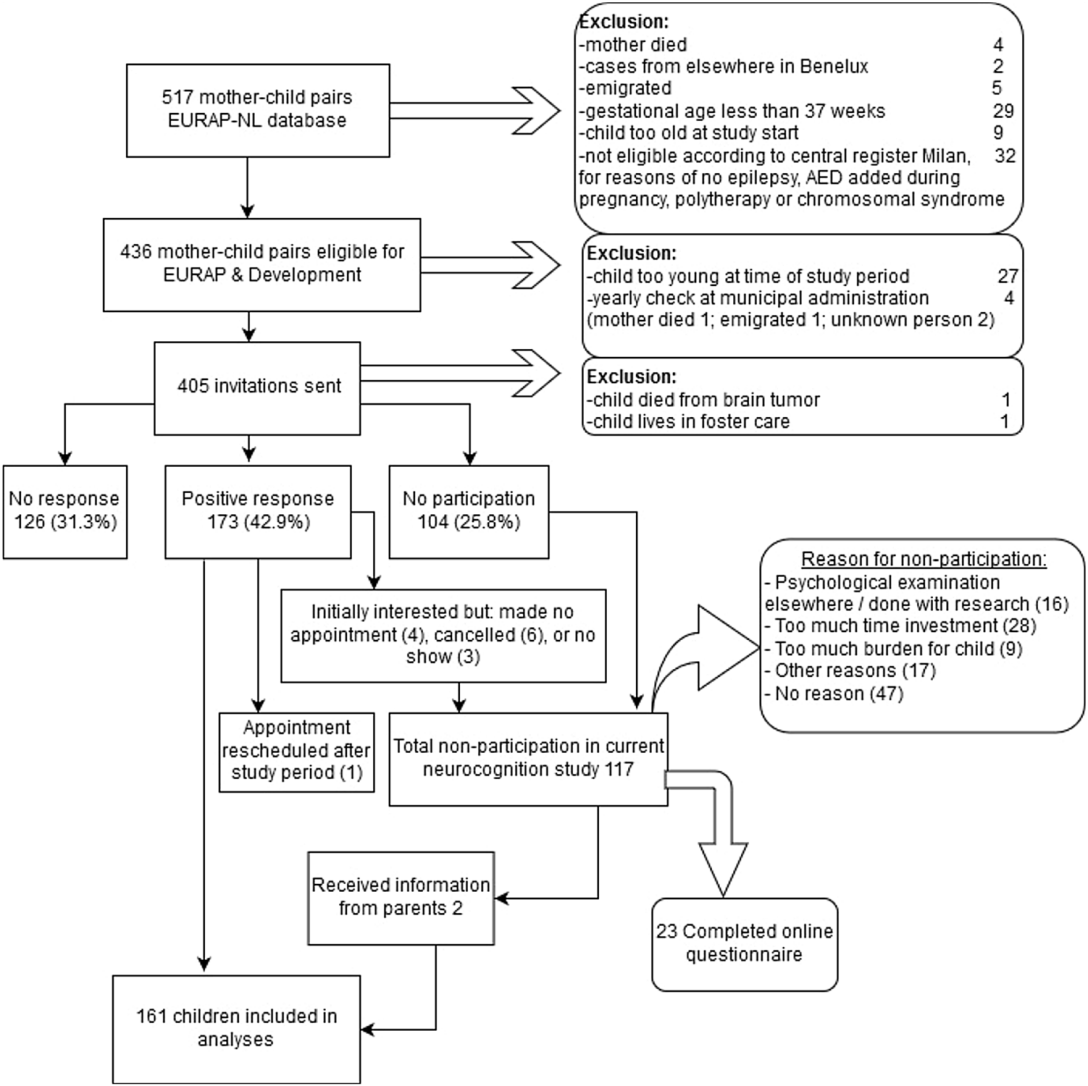
Flowchart inclusion Dutch EURAP & Development study

Four hundred and five invitations to participate were sent with 173 positive responses received (42.9%). 117 families declined participation (28.9%), and 126 did not respond (31.3%) (Fig. 1). Between January 2015 and February 2018 one hundred and sixty-one children of 139 mothers (one pair of twins and 21 pairs of siblings) were included for this neurocognition study (mean age 82 months; range 72–97 months—one child was 8.1 years at assessment because of a rescheduled appointment). This was approximately 40% of the original mother–child pairs who participated in EURAP-NL. The inclusion rate per AED was VPA 37%, CBZ 28%, LTG 51%, and LEV 36%.

For the outcome variables there were few missing values (Tables 2, 3). Extra tasks to the test protocol (design fluency, word generation, visual attention, and phonological processing) were not assessed in all children, due to lack of time or motivation. Some tasks were available only for certain ages (statue and auditory synthesis at 6 years only; phoneme deletion from 6.5 years).

**Table 2:** Means and standard deviations of full scale, verbal and performance intelligence and processing speed, and percentage of children scoring below 85, by AED group

WISC-III-NL	Sample size	VPA (22)	CBZ (32)	LTG (82)	LEV (25)	*p* value
		*M* (SD) range No. (%) < 85	*M* (SD) range No. (%) < 85	*M* (SD) range No. (%) < 85	*M* (SD) range No. (%) < 85	
FSIQ	161	103.2 (14.8) 73–138 1 (4.5)	105.3 (13.7) 70–125 3 (9.4)	109.2 (15.0) 71–148 3 (3.7)	110.8 (14.8) 77–136 1 (4.0)	0.188^a^
VIQ	161	100.6 (14.9) 70–126 4 (18.2)	106.2 (14.2) 86–138 0 (0)	109.7 (15.7) 64–150 6 (7.3)	114.0 (13.1) 88–140 0 (0)	0.014^a^
PIQ	161	105.3 (17.0) 77–140 3 (13.6)	102.8 (15.5) 62–127 4 (12.5)	106.0 (14.9) 77–146 6 (7.3)	104.4 (14.8) 73–129 3 (12.0)	0.796^a^
PSI	153	107.4 (18.6) 72–143 3 (14.3)	108.7 (12.1) 75–137 1 (3.3)	111.0 (14.4) 75–140 3 (3.9)	111.2 (16.7) 69–142 1 (4.0)	0.722^a^

Means are unadjusted for covariates. Test mean is 100 with a standard deviation of 15. IQ below 85 is classed as a below average performance

*WISC-III-NL* [16] Wechsler Intelligence Scale for Children-third edition, *FSIQ* full scale intelligence, *VIQ* verbal intelligence, *PIQ* performance intelligence, *PSI* processing speed index, *VPA* valproate, *CBZ* carbamazepine, *LTG* lamotrigine, *LEV* levetiracetam, *M* mean, *SD* standard deviation

^a^Analysis of variance (normal distribution)

**Table 3:** Means and standard deviations by AED group for specific neurocognitive outcomes

NEPSY-II-NL^a^, Tea-CH^a^, PPVT-III-NL^b^, Lindeboom^a^	Sample size	VPA (20)	CBZ (32)	LTG (82)	LEV (25)	*p* value
Attention and executive functioning						
Auditory attention^g^	157	9.8 (2.2)	9.8 (2.7)	9.4 (2.6)	9.9 (2.4)	0.790^f^
Response set^g^	148	8.3 (3.1)	8.9 (2.5)	9.1 (2.2)	9.2 (2.8)	0.762^f^
Inhibition—naming total errors^g^	156	8.5 (2.3)	8.6 (2.4)	8.8 (2.3)	9.3 (2.5)	0.526^f^
Inhibition—naming time score	156	9.9 (3.1)	11.0 (2.3)	11.4 (2.1)	11.5 (2.4)	0.069^d^
Inhibition—inhibition total errors^g^	156	8.5 (2.2)	8.8 (2.4)	8.9 (2.5)	9.5 (2.2)	0.593^f^
Inhibition—inhibition time score^g^	156	8.2 (2.5)	8.9 (2.3)	8.9 (1.8)	9.1 (1.9)	0.501^f^
Statue^g^	117	6.8 (2.0)	8.6 (2.5)	9.0 (3.2)^h^	8.6 (2.9)	0.052^f^
Design fluency	100	9.2 (3.1)^h^	11.1 (3.5)^h^	11.2 (3.1)^h^	11.4 (3.2)^h^	0.234^d^
Visual attention^a^	102	11.1 (3.4)	11.7 (3.1)^h^	11.3 (2.6)^h^	10.4 (3.1)	0.610^d^
Language skills						
Comprehension of instructions	157	9.2 (2.1)	10.6 (2.3)	11.2 (3.0)	12.0 (3.5)	0.004^e^
Speeded naming time score^g^	155	8.2 (2.7)	9.2 (2.5)	9.3 (2.4)	9.4 (2.5)	0.313^f^
Speeded naming total correct^g^	155	7.2 (3.1)	8.6 (3.3)	8.7 (3.1)	7.8 (3.2)	0.190^f^
Word generation	118	8.9 (2.9)	10.0 (3.1)^h^	10.9 (2.5)^h^	10.7 (3.2)	0.075^d^
Verbal fluency^a^—time score	157	9.7 (4.6)	8.5 (4.3)	11.1 (4.4)	10.3 (4.6)	0.048^d^
Vocabulary—WBQ^b^	157	105.7 (11.3)	110.1 (10.8)	111.0 (13.2)	114.9 (9.8)	0.105^d^
Auditory synthesis^c^	75	7.3 (1.5)^h^	7.0 (2.0)^h^	7.2 (2.0)^h^	8.3 (1.8)^h^	0.118^d^
Phoneme deletion^a^	46	9.7 (4.1)^h^	10.3 (2.9)^h^	10.6 (2.4)^h^	9.8 (1.5)^h^	0.857^d^
Memory and learning						
Memory for faces	158	9.2 (4.0)	11.3 (3.4)	10.6 (2.6)	9.9 (3.5)	0.211^e^
Memory for faces delayed	157	10.6 (4.7)	12.1 (3.0)	11.2 (2.5)	11.6 (2.7)	0.427^e^
Memory for names	155	8.8 (3.0)	8.7 (2.7)	8.9 (2.7)	9.8 (2.9)	0.448^d^
Memory for names delayed	155	7.7 (3.9)	8.1 (3.6)	8.1 (3.7)	8.8 (4.5)	0.778^d^
Narrative memory—free and cued^g^	156	9.2 (2.2)	9.1 (2.4)	9.4 (2.2)	9.5 (2.4)	0.852^f^
Fine motor skills						
Imitating hand positions	155	10.6 (3.0)	11.2 (3.0)	11.1 (2.5)	11.5 (3.2)	0.762^d^
Fingertip tapping—repetition DH	154	12.0 (1.2)	12.4 (1.3)	11.9 (2.0)	12.2 (1.9)	0.556^f^
Fingertip tapping—repetition NDH	154	11.7 (1.3)	11.7 (1.1)	11.5 (1.9)	11.8 (1.4)	0.946^f^
Fingertip tapping—series DH	151	9.0 (3.3)	10.5 (1.7)	10.6 (2.1)	10.6 (2.2)	0.201^f^
Fingertip tapping—series NDH^g^	151	8.4 (3.2)	9.6 (2.3)	9.3 (2.3)	9.4 (2.5)	0.624^f^
Visuomotor precision time score^g^	157	9.1 (2.6)	7.9 (2.5)	8.0 (2.6)	8.8 (2.6)	0.245^f^
Visuomotor precision total errors^g^	157	6.5 (2.6)	8.4 (2.1)	8.7 (2.5)	7.5 (2.3)	0.004^f^
Visuospatial skills						
Arrows	155	11.1 (3.8)	12.2 (3.3)	12.5 (3.1)	12.3 (3.0)	0.358^d^
Design copying^g^	156	8.3 (2.5)	9.3 (2.2)	9.2 (1.9)	9.0 (2.2)	0.293^d^

Means are unadjusted for covariates. Lindeboom [22]—verbal fluency task, Auditory Synthesis from “language test for children” [23]. Phoneme Deletion from “Dyslexia Screening Test” [24]

*NEPSY-II-NL* [19] developmental neuropsychological assessment—second edition, *Tea-CH* [20] Test of Everyday Attention for Children, *PPVT-III-NL* [21] Peabody Picture Vocabulary Test—third edition, *VPA* valproate, *CBZ* carbamazepine, *LTG* lamotrigine, *LEV* levetiracetam, *DH* dominant hand, *NDH* non-dominant hand

^a^Test mean is 10 with a standard deviation of 3. Standard score 8–12 is average

^b^Test mean is 100 with a standard deviation of 15. IQ scores between 90 and 110 are interpreted as average

^c^Decile scores

^d^Analysis of variance (normal distribution)

^e^Welch (no homogeneous group)

^f^Kruskal–Wallis (skewed)

^g^Original percentile score, converted to standard scores [19]; standard score between 8 and 12 are average

^h^More than 25% missing

Children from the four AED-exposed groups were comparable across most demographic variables (Table 1). Significant differences were found in children exposed to nicotine in the first trimester, with highest rates seen for children from the VPA-exposed group (27%). The mothers of children exposed to VPA were also slightly but significantly older at the child’s birth. Mothers who used LTG were significantly more likely to have consumed alcohol during the first trimester (32%). Epilepsy type differed significantly between groups. Mothers who used VPA significantly more often had generalized epilepsy (73%) while mothers who used CBZ significantly more often had focal epilepsy (88%). Children who were exposed to VPA had significantly more congenital malformations (23%) than those who were exposed to CBZ (13%), LTG (5%) or LEV (4%).

Parent-reports showed that the majority of children attended mainstream schools (Table 1). Many children, however, received additional support at school, speech therapy, or physiotherapy. VPA exposed children tended to have higher frequencies of additional educational needs (36%) but this was not significant (*p* = 0.099, Fisher test). No significant differences were found for rates of children with developmental delay according to type of AED, as per parent reported (*p* = 0.088, Fisher test).

### The nature and severity of neurocognitive development

Across the AED groups, VPA-exposed children had the lowest unadjusted mean scores for full scale intelligence (FSIQ), Verbal intelligence (VIQ) and processing speed (PSI) (Table 2), and across most specific neurocognitive domains (Table 3).

Compared to the norms, all children generally scored within the average range on neurocognitive outcome measures, except for VPA-exposed children, who performed below average on the tasks of statue and memory for names delayed. VPA-exposed children and LEV-exposed children also performed below average on speeded naming (total correct) and visuomotor precision (total errors). CBZ-exposed children performed below average on visuomotor precision (time score). On other outcome measures children performed at least low on average (standard score 8) and sometimes above average (Tables 2, 3).

**Fig. 2:**
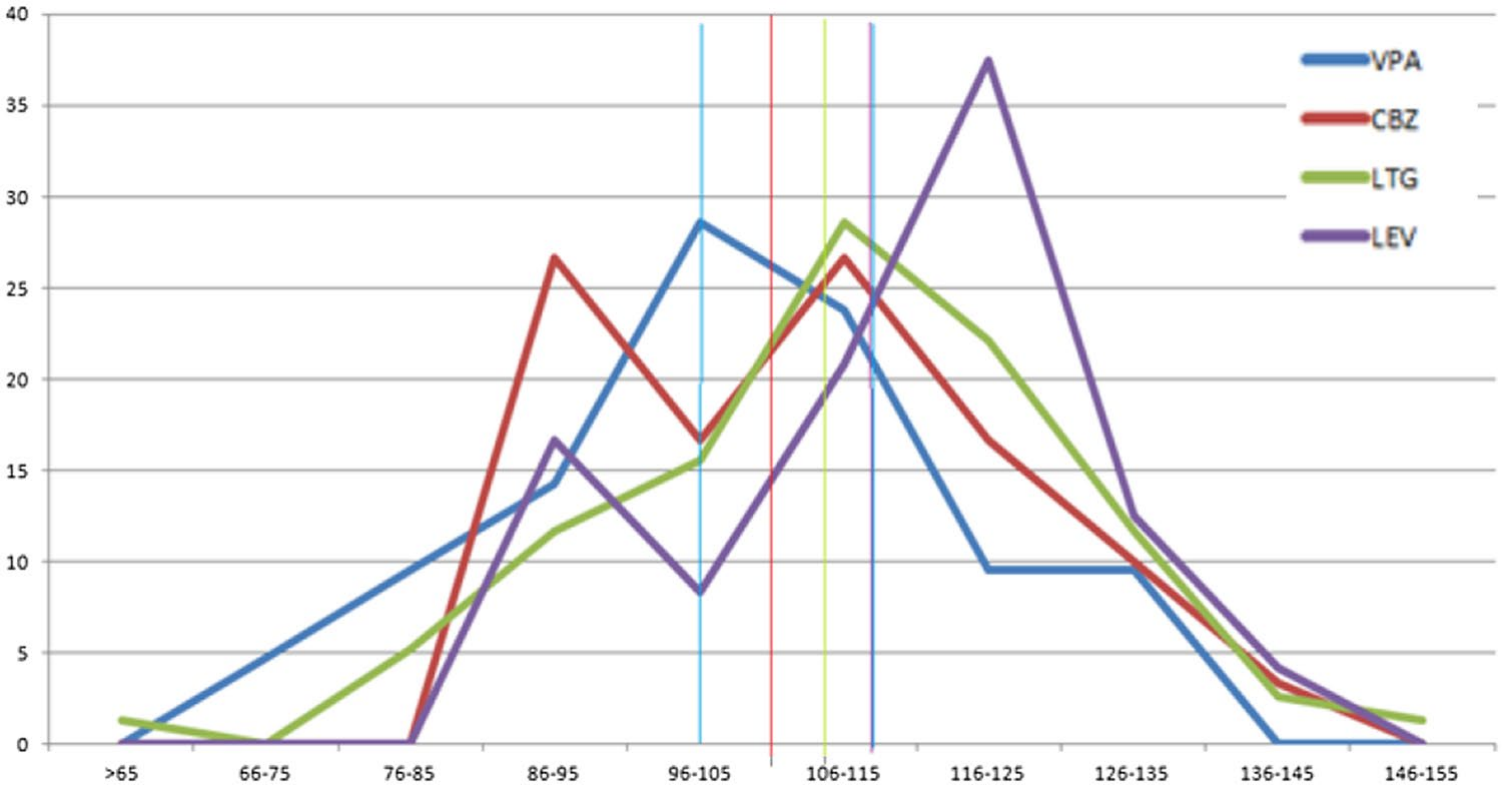
Distribution of verbal IQ scores of exposed children across the four AED groups

Children exposed to VPA scored lower on verbal intelligence. The mean score on verbal IQ is average but the distribution of verbal IQ scores appears somewhat shifted to the left, indicating overall lowered scores when contrasted to the other AED groups (Fig. 2). Similar distributions were found for the other neurocognitive outcome measures. LEV-exposed children scored above average on verbal intelligence, comprehension of instructions and vocabulary.

Because maternal IQ is an important confounder for child IQ, we also calculated outcome variable means adjusted for maternal IQ, but this gave similar results (Table 4). Adjusted verbal IQ was for VPA 100.5 (SE 2.9; 95% CI 94–106), for CBZ 107.9 (SE 2.5; 95% CI 103–113), for LTG 109.6 (SE 2.5; 95% CI 107–113), and for LEV 112.3 (SE 2.8; 95% CI 107–118).

**Table 4:** Adjusted means of full scale, verbal and performance intelligence and processing speed

WISC-III-NL	Sample size	VPA (22)		CBZ (32)		LTG (82)		LEV (25)	
		Mean^a^ (SE)	95% CI	Mean^a^ (SE)	95% CI	Mean^a^ (SE)	95% CI	Mean^a^ (SE)	95% CI
FSIQ	161	103.1 (2.9)	97–109	106.9 (2.4)	102–112	109.1 (1.5)	106–112	109.2 (2.8)	104–115
VIQ	161	100.5 (2.9)	95–106	107.9 (2.5)	103–113	109.6 (1.5)	107–113	112.3 (2.9)	107–118
PIQ	161	105.2 (3.2)	99–111	104.0 (2.6)	99–109	105.9 (1.6)	103–109	103.3 (3.0)	97–109
PSI	153	107.8 (3.1)	102–114	109.9 (2.6)	105–115	110.8 (1.6)	108–114	109.8 (2.9)	104–116

*WISC-III-NL* [16] Wechsler Intelligence Scale for Children-third edition, *FSIQ* full scale intelligence, *VIQ* verbal intelligence, *PIQ* performance intelligence, *PSI* processing speed index, *VPA* valproate, *CBZ* carbamazepine, *LTG* lamotrigine, *LEV* levetiracetam, *SE* standard error, *CI* confidence interval

^a^Means are adjusted for maternal IQ (mean 104.65)

LEV-exposed children frequently had a disharmonic profile—meaning a significant difference of more than 16 points between VIQ and PIQ—in favor of verbal skills (seven had VIQ > PIQ vs one who had VIQ < PIQ). In VPA-exposed children it was the opposite with more disharmonic profiles in favor of performance skills (2 VIQ > PIQ vs 4 VIQ < PIQ). For CBZ- and LTG-exposed children this was 6 vs 5 and 20 vs 10 respectively.

### Comparison between children exposed to different antiepileptic drug types

After controlling for maternal IQ, standardized dose and VPA-dose, multiple regression analyses showed that VPA-exposed children scored significantly lower on FSIQ than those exposed to LTG, and significantly lower on VIQ than CBZ-, LTG-, and LEV-exposed children. No significant differences were found for PIQ or PSI (Table 5).

**Table 5:** Multiple regression analyses of intelligence outcomes with VPA as reference group

WISC-III-NL	VIQ			PIQ			FSIQ			PSI		
	*B* (SE)	95% CI	*p* value	*B* (SE)	95% CI	*p* value	*B* (SE)	95% CI	*p* value	*B* (SE)	95% CI	*p* value
Constant	55.9 (8.7)	38.7–73.0	0.000	75.2 (9.4)	56.7–93.7	0.000	60.3 (8.6)	43.3–77.3	0.000	71.9 (9.2)	53.6–90.2	0.000
CBZ	9.1 (4.0)	1.3–17.0	0.023*	0.1 (4.3)	− 8.3–8.6	0.973	5.6 (3.9)	− 2.2–13.4	0.157	5.1 (4.2)	− 3.3–13.5	0.229
LTG	10.3 (3.5)	3.4–17.3	0.004**	2.3 (3.8)	− 5.3–9.8	0.551	7.5 (3.5)	0.6–14.4	0.033*	6.3 (3.8)	− 1.1–13.7	0.097^†^
LEV	13.4 (4.2)	5.2–21.6	0.002**	− 0.6 (4.5)	− 9.5–8.3	0.901	7.7 (4.1)	− 0.4–15.8	0.064†	4.9 (4.4)	− 3.9–3.6	0.275
Maternal IQ	0.4 (0.1)	0.3–0.6	0.000**	0.3 (0.08)	0.1–0.4	0.001**	0.4 (0.08)	0.2–0.5	0.000**	0.3 (0.08)	0.2–0.5	0.000**
Dose	0.02 (0.02)	− 0.02–0.05	0.417	− 0.01 (0.02)	− 0.05–0.03	0.677	0.005 (0.02)	− 0.03–0.04	0.774	− 0.01 (0.01)	− 0.05–0.02	0.476
VPA Dose	− 0.2 (0.1)	− 0.5–0.02	0.071	− 0.1 (0.1)	− 0.4–0.1	0.284	− 0.2 (0.1)	− 0.4–0.3	0.081	− 0.2 (0.1)	− 0.5–0.02	0.065

Dose was standardized based on the formula: [100 × ((dose 1st trimester − median AED dose)/median AED dose)]

*WISC-III-NL* Wechsler Intelligence Scale for Children-third edition, *FSIQ* full scale intelligence, *VIQ* verbal intelligence, *PIQ* performance intelligence, *PSI* processing speed index, *VPA* valproate, *CBZ* carbamazepine, *LTG* lamotrigine, *LEV* levetiracetam, *B* unstandardized coefficients, *SE* standard error, *CI* confidence interval

*p < 0.05

**p < 0.01

^†^*p* < 0.10

On specific neurocognitive domains, VPA-exposed children performed significantly lower on the following sub-scores of attention and executive functions: [statue: VPA < CBZ, LTG, LEV; inhibition naming (time score): VPA < CBZ, LTG, LEV; design fluency: VPA < LTG], language skills [comprehension of instruction: VPA < CBZ, LTG, LEV; speeded naming (total correct): VPA < LTG; word generation: VPA < LTG; vocabulary: VPA < LEV], memory and learning [memory for faces and memory for faces delayed: VPA < CBZ], fine motor skills [fingertip tapping series dominant hand: VPA < LTG; visuomotor precision (total errors): VPA < CBZ, LTG], and visuospatial skills [arrows: VPA < LTG; design copying: VPA < CBZ and LTG] (see supplemental material e-Table 5).

Additional analyses with LTG-exposed group as reference and maternal IQ, standardized dose and LTG dose as confounders, revealed virtually no significant differences between children exposed to LTG and LEV or LTG and CBZ (e-Table 6). LTG-exposed children only performed significantly better than LEV-exposed children on Visuomotor Precision (total errors; − 1.3, CI − 2.4 to − 0.2, *p* = 0.022) and achieved a significantly higher score on the Verbal Fluency task (Lindeboom) compared to children exposed to CBZ (− 2.3, CI − 4.2 to − 0.4, *p* = 0.017).

### Antiepileptic drug dose

For children exposed to LEV, LTG or CBZ no dose–effect was found. The effect of VPA dose was significant for a number of outcome measures (Statue (*p* = 0.032), phoneme deletion (*p* = 0.017), memory for names (*p* = 0.032), memory for names delayed (*p* = 0.029), and narrative memory (*p* = 0.025)). The association between child IQ and VPA-dose was nonsignificant. It made no difference whether we examined first trimester dose or third trimester dose, for both we found no significant relationship between cognitive outcome measures and dose.

### Confounding factors

Epilepsy type differed significantly between the different types of AED used. Mothers using VPA mainly had generalized epilepsy which could suggest confounding by indication. Epilepsy type was, however, not associated with the outcome measures. The presence of convulsions during pregnancy was also not associated with the outcome measures. The presence of congenital malformations was associated with some of the outcome measures (e.g., FSIQ), but after controlling for type of AED (interaction term between malformations and AED), this was no longer significant. As expected, higher maternal IQ was associated with improved child performance on intelligence and specific neurocognitive measures (Table 5; e-Table 5). Maternal education, which we used as proxy for social economic status (SES), was also associated with child outcome measures, but because they are intercorrelated, only maternal IQ was included in the analyses. Other potential confounders have been thoroughly examined but were not found to be associated with the outcome measures (see supplemental material, e-Table1a).

### Sensitivity analyses

Some children shared the same mother, father, and family environment, which may cause dependency between outcome measures. Multilevel analyses, however, did not suggest significant dependency. To confirm this, we also conducted a sensitivity analysis with only one child from each family (*n* = 139). This yielded similar results. Sensitivity analyses with only children included before 16 weeks of gestation (*n* = 117) showed also similar results (not shown, available on request).

## Discussion

This study showed differences in neurocognitive functioning between children exposed prenatally to four common AED monotherapies. Consistent with previous observational studies [5, 8, 13, 27–29], VPA-exposed children performed less-well in all neurocognitive domains than children exposed to CBZ, LTG, or LEV, especially on language skills (9–13 points lower on VIQ). A direct comparison, between the largest group of school-aged LTG-exposed children and LEV- or CBZ-exposed children, showed virtually no significant differences, after controlling for potential confounders. Children exposed to LTG, CBZ or LEV performed at average to above average levels on intelligence and specific neurocognitive functions. This is consistent with previous studies finding no or fewer problems after exposure to CBZ, LTG or LEV [5, 8, 13, 27].

We noticed that LEV-exposed children more often had disharmonic profiles which were opposite to VPA-exposed children’s profiles. LEV-exposed children appeared to have higher developmental scores than VPA-exposed children, indicating fewer problems. However, no increased intelligence was found in the previous reported study on school-aged LEV-exposed children [8]. Our group of children exposed to LEV was relatively small, so new studies using larger samples are needed to examine long-term functioning in LEV-exposed children.

We found no dose–effect for LEV, LTG or CBZ. Within the VPA-exposed group, the dose–effect for cognition (intelligence) was nonsignificant, while some other neurocognitive measures showed a dose effect for VPA. Other studies have reported that the risk of VPA is dose-related. Earlier studies indicated that differences in neurocognition cause problems at a dose of 800–1000 mg of valproate [5, 27], differences at lower doses have also been found (< 400 mg) [30], with higher doses giving rise to more problems [5, 7, 27]. Our negative finding may be due to the small sample size of the VPA-exposed group.

The dose in the third trimester appears to be particularly important for cognitive development [31]. Correlation analyses with third trimester dose showed no differences compared to first trimester dose. In particular the dose of LTG was often increased (Table 1). It is known however, that the clearance of LTG changes during pregnancy because of pharmacokinetics [32]. It would therefore be more reliable to use AED blood levels, but those were not available. We examined the median AED dose within the first trimester instead. However, we are aware that this has limitations, also because we did not have access to adherence data.

Frequent convulsions during pregnancy have previously been associated with reduced cognitive functions in the child [33], but this has not been found in other studies [5, 13]. In our study there was no significant difference between mothers from the four AED groups regarding the occurrence of tonic–clonic seizures. No associations were found between tonic–clonic seizures and outcome measures. We conducted a post hoc analysis into tonic–clonic seizure frequency (with five or more seizures), but this did not reveal differences. There was also no association found for type of epilepsy.

Pre-conceptional folate has been associated with higher intelligence scores in children of mothers with epilepsy [5], and was recently associated with a reduced risk of autistic traits [34]. We have seen no associations between preconception folic acid use and outcomes.

The number of children with congenital malformations differed significantly between the different types of AED, with VPA-exposed children most affected. The rate of malformations within the VPA and CBZ exposed group showed to be higher than typically reported within pregnancy registers [7, 12]. When type of AED and its interaction with presence or absence of malformations was controlled for, the association between malformations and neurocognitive outcomes was, however, no longer significant.

Positive associations seem to exist between breastfeeding and outcome measures [35, 36]. In our study no such association was found. This may be due to the rather small proportion of breast-fed children in our study (16–38%). Over the years (2007–2011), a slight increase in breastfeeding can been seen in our study, but most women had been advised not to breastfeed their children. For mothers with epilepsy who wish to breastfeed, the benefits are usually considered to outweigh the risks [37]. This calls for providing more accurate information about breastfeeding and AEDs to women with epilepsy [38].

This study has several strengths. First, the prospective design of the study with recruitment of mother–child pairs through the national pregnancy register (EURAP-NL). Secondly, children and their mothers and fathers were extensively assessed by assessors blinded to AED exposure type, using standardized neuropsychological measures [31]. Thirdly, a major methodological strength was the rigorous control for potential confounders, including AED-dose and maternal IQ [39]. Finally, the large size of the LTG-exposed group and inclusion of LEV-exposed children allowed us to obtain more insights into associations between these increasingly prescribed AEDs and child outcomes [8].

There also are limitations to our study. The statistical threshold (*p* < 0.05) used for the multiple regression analyses was uncorrected for the multitude of analyses. This gives a risk of probability capitalization. We consider, however, that the number of significantly found p-values was greater than could be expected on the basis of 5% chance. In addition, the mean differences were large across the outcome measures on the whole and support the statistical findings thereby reducing the likelihood that the findings were by chance,

We also note that pregnancy registers only reach part of the women with epilepsy. This may limit the generalization of the results to the population of mothers with epilepsy. Generalization may also be limited due to the relatively high educational level of mothers across all four exposure groups. This may mainly explain the average to above-average IQ in the children. Future studies should aim to obtain more inclusive recruitment of families from lower educational levels.

The Flynn effect may also explain in part the apparently average or above average functioning of the children. The Flynn effect is the increase in intelligence scores within a population over the years [40]. The Flynn effect may have been enhanced using the WISC-III-NL [16]. In other countries the WISC-IV or WISC-V are used, but in The Netherlands these WISC-versions were not available at the start of the study period. In other countries using a wide range of different cognitive tests and test versions, children of mothers with epilepsy score average [5, 8, 27], or below average [28, 41, 42].

The group of mothers that participated had in general well controlled seizures. This might also have caused a selection bias. Mothers with well controlled seizures may have been able to attend the assessment with more ease. We partly managed to overcome this problem because we could assess mother–child pairs at home and at regional centers.

Because the purpose of this study was a comparison between different types of AEDs monotherapy, a control group of non-exposed children was not included. Children who were exposed to AED polytherapy were also not included. More research is needed to answer related questions about respectively a comparison with non-exposed children or children exposed to different polytherapy combinations [6].

The number of children within each AED group differed, with a larger number of children exposed to LTG and a relatively smaller group of children exposed to VPA, LEV, and CBZ. The inclusion rate of children for the four AEDs, however, was similar (approximately 40%), but lowest for CBZ and highest for LTG. Our inclusion rate is comparable with other prospective observational studies [8], but not with studies with multiple follow-ups from early age on [5, 27]. The lower number of children exposed to VPA can be explained by a steady decline over the years in The Netherlands of the number of women using VPA during pregnancy paralleled by an increase in LTG or LEV monotherapy. The use of VPA is declining but for some women with epilepsy other AEDs are not a suitable choice [43]. VPA is also increasingly prescribed for psychiatric disorders [44]. More insight into contributing factors to vulnerability in VPA-exposed children is warranted. Based on current reproductive toxicological knowledge, VPA prescriptions for epilepsy in women of reproductive age may be replaced by prescriptions of AEDs with lower teratogenic profile, such as LEV or LTG [45, 46]. Meanwhile, further confirmation of these findings by future studies of neurocognitive functioning in LEV- and LTG-exposed children is required. Collaborative studies and pooling of data may facilitate this.

In summary, VPA-exposed children performed worse than children exposed to CBZ, LTG and LEV, while few differences were found within a comparison between the three other AEDs. This has implications for pre-pregnancy counseling. To date, healthcare for women with epilepsy has paid little attention to continued monitoring of children of mothers with epilepsy [47]. It is essential that children of mothers with epilepsy be followed over time. Developmental problems may then be detected in a timely manner and treated accordingly. In this study we did not consider the role of active maternal epilepsy during infant and child development. It will be worthwhile examining these aspects in future studies as they are of importance for developing interventions [48]. This may ultimately enhance the quality of life of children who have been exposed to AEDs in utero, their mothers, and their families.

## Electronic supplementary material

Below is the link to the electronic supplementary material.

Supplementary file1 (PDF 276 kb)
