# Supplementary material for: Neurocognition after prenatal levetiracetam, lamotrigine, carbamazepine or valproate exposure
Source: J Neurol. 2020 Feb 28;267(6):1724–36. doi: 10.1007/s00415-020-09764-w (PMC7293688; doi:10.1007/s00415-020-09764-w)
Supplement: Supplementary file 1 — Supplementary file1 (PDF 276 kb) [file 415_2020_9764_MOESM1_ESM.pdf]

Supplementary material, Journal of Neurology

Article: Neurocognition after prenatal levetiracetam, lamotrigine, carbamazepine or valproate exposure

Authors: Yfke Huber-Mollema, PhD[1,2]; Loretta van Iterson PhD[1]; Frans J. Oort, PhD[2]; Dick Lindhout, MD, PhD[1,3]; Roos Rodenburg, PhD[1,2]

[1]Stichting Epilepsie Instellingen Nederland (SEIN), Heemstede, The Netherlands

[2]Research Institute of Child Development and Education, University of Amsterdam, The Netherlands

[3]Department of Genetics, University Medical Center Utrecht, The Netherlands

Corresponding author: Roos Rodenburg, H.R.Rodenburg@uva.nl

e-Table 1a

Bivariate correlations between potential confounders (a - p) and cognitive outcome measures

	VIQ	PIQ	FSIQ	PSI
a. Maternal IQ	,389**	,257**	,382**	,300**
b. Type of maternal epilepsy	-,016	,035	,010	-,019
c. Tonic-clonic seizures during pregnancy	,093	,028	,074	-,045
d. Use of periconceptional folic acid	-,013	,047	,016	,001
e. Alcohol exposure during first trimester	-,052	-,047	-,057	-,042
f. Alcohol exposure during second or third trimester	-,065	-,020	-,045	-,033
g. Nicotine exposure during first trimester	-,083	-,087	-,103	,016
h. Nicotine exposure during second or third trimester	-,122	-,121	-,145	-,103
i. Breastfeeding	-,126	-,049	-,109	-,072
j. Maternal age at delivery	-,012	,017	,002	,007
k. Maternal education	,384**	,294**	,404**	,249**
l. Gestational age	,077	,022	,055	,094
m. Gender	-,041	-,037	-,046	,101
n. Age at assessment	-,029	,055	,018	-,051
o. Presence or absence of congenital malformations	-,124	-,167*	-,175*	-,344**
p. Time of inclusion in the EURAP-NL database	,055	,100	,089	-,006

Cognitive outcome measures: VIQ = verbal intelligence, PIQ = performance intelligence, FSIQ = full scale intelligence, PSI = processing speed index.

Potential confounders: a. Maternal IQ measured with WAIS-III-NL; b. generalized epilepsy (yes, no, dichotomous); c. Tonic-clonic seizures during pregnancy (yes, no, dichotomous); d. Periconceptional use of folic acid (yes, no dichotomous); e. Alcohol use during first trimester ((yes, no dichotomous); f. Alcohol use during second or third trimester (yes, no dichotomous); g. Nicotine use during first trimester (yes, no dichotomous); h. Nicotine use during second or third trimester (yes, no dichotomous); i. Breastfeeding (yes, no dichotomous); j. Maternal age at delivery (years, interval); k. Maternal education: higher educated (yes/no, dichotomous); l. Gestational age (weeks, interval); m. Gender (boy, girl, dichotomous); n. Age of child at time of study (months, interval); o. Presence or absence of congenital malformations (yes, no dichotomous); p. Time of inclusion, prospective (yes, no, dichotomous).

* $p < .05$ ** $p < .01$

e-Table 5

Multiple regression analyses of specific neurocognitive outcomes with VPA as reference group

	Attention and Executive functioning											
	Auditory Attention			Response Set			Statue			Design Fluency		
	B (SE)	95% CI	*p* Value	B (SE)	95% CI	*p* Value	B (SE)	95% CI	*p* Value	B (SE)	95% CI	*p* Value
Constant	4.4 (1.6)	1.3 to 7.6	.006	3.1 (1.6)	0.02 to 6.2	.049	3.6 (2.1)	-0.5 to 7.7	.082	2.3 (2.5)	-2.6 to 7.2	.352
CBZ	0.2 (0.7)	-1.2 to 1.6	.780	0.5 (0.7)	-0.9 to 2.0	.445	2.3 (0.9)	0.5 to 4.2	.016*	2.1 (1.1)	-0.1 to 4.4	.064†
LTG	-0.4 (0.6)	-1.7 to 0.8	.494	0.6 (0.6)	-0.7 to 1.8	.385	2.5 (0.8)	0.8 to 4.1	.004*	2.0 (1.0)	0.04 to 4.0	.046*
LEV	-0.2 (0.8)	-1.7 to 1.3	.826	0.5 (0.8)	-1.0 to 1.9	.546	2.1 (1.0)	0.1 to 4.1	.037*	1.9 (1.2)	-0.5 to 4.2	.115
Maternal IQ	0.05 (0.01)	0.02 to 0.08	.000**	0.05 (0.01)	0.03 to 0.08	.000**	0.03 (0.02)	-0.01 to 0.06	.152	0.07 (0.02)	0.02 to 0.1	.003**
Dose	-0.001 (0.003)	-0.006 to 0.006	.994	-0.002 (0.003)	-0.008 to 0.005	.572	0.003 (0.004)	-0.005 to 0.01	.468	-0.006 (0.005)	-0.02 to 0.004	.262
VPA Dose	0.01 (0.02)	-0.03 to 0.06	.597	0.03 (0.02)	-0.02 to 0.07	.264	-0.06 (0.03)	-0.1 to -0.005	.032*	0.006 (0.04)	-0.06 to 0.08	.861
	Inhibition – naming total errors			Inhibition – naming time score			Inhibition- inhibition total errors			Inhibition – inhibition time score		
	B (SE)	95% CI	*p* Value	B (SE)	95% CI	*p* Value	B (SE)	95% CI	*p* Value	B (SE)	95% CI	*p* Value
Constant	8.1 (1.5)	5.1 to 11.1	.000	5.2 (1.5)	2.4 to 8.1	.000	7.0 (1.5)	4.0 to 10.0	.000	6.1 (1.3)	3.6 to 8.6	.000
CBZ	0.04 (0.7)	-1.3 to 1.4	.957	1.3 (0.7)	0.03 to 2.6	.046*	0.3 (0.7)	-1.1 to 1.6	.707	0.8 (0.6)	-0.4 to 1.9	.187
LTG	0.2 (0.6)	-1.0 to 1.4	.731	1.5 (0.6)	0.4 to 2.7	.011*	0.1 (0.6)	-1.1 to 1.4	.815	0.6 (0.5)	-0.4 to 1.7	.229
LEV	0.7 (0.7)	-0.7 to 2.2	.314	1.4 (0.7)	0.01 to 2.8	.049*	0.8 (0.7)	-0.6 to 2.3	.250	0.8 (0.6)	-0.4 to 2.1	.171
Maternal IQ	0.004 (0.01)	-0.02 to 0.03	.745	0.04 (0.01)	0.02 to 0.07	.001**	0.01 (0.01)	-0.01 to 0.04	.282	0.02 (0.01)	-0.003 to 0.04	.082
Dose	0.000 (0.003)	-0.006 to 0.006	.962	0.001 (0.03)	-0.005 to 0.007	.747	0.005 (0.003)	-0.001 to 0.01	.085	0.004 (0.003)	-0.001 to 0.009	.119
VPA Dose	0.002 (0.02)	-0.04 to 0.04	.921	-0.02 (0.02)	-0.06 to 0.02	.381	-0.009 (0.02)	-0.05 to 0.03	.667	-0.01 (0.02)	-0.05 to 0.02	.447

Note. VPA: valproate; CBZ; carbamazepine; LTG: lamotrigine; LEV: levetiracetam.

Dose was standardized based on the formula: [100 x ((dose 1st trimester– median AED dose) / median AED dose)].

B = unstandardized coefficients, SE = standard error, CI = Confidence Interval.

†< .10 *$p < 0.05$ ** $p < 0.01$

to be continued (1 of 3)

Continued (2 of 3)

	Language Skills											
	Comprehension of Instruction			Speeded Naming time score			Speeded Naming total correct			Word Generation		
	B (SE)	95% CI	*p* Value	B (SE)	95% CI	*p* Value	B (SE)	95% CI	*p* Value	B (SE)	95% CI	*p* Value
Constant	2.2 (1.7)	-1.2 to 5.7	.201	4.4 (1.6)	1.3 to 7.5	.005	4.0 (2.0)	-0.05 to 8.0	.053	3.3 (2.0)	-0.7 to 7.2	.105
CBZ	1.7 (0.8)	0.1 to 3.3	.033*	1.2 (0.7)	-0.2 to 2.6	.093†	1.8 (0.9)	-0.04 to 3.6	.055†	1.5 (0.9)	-0.3 to 3.3	.109
LTG	2.0 (0.7)	0.6 to 3.4	.005**	1.1 (0.6)	-0.2 to 2.3	.091†	1.8 (0.8)	0.1 to 3.4	.033*	2.1 (0.8)	0.5 to 3.7	.011*
LEV	2.5 (0.8)	0.8 to 4.1	.004**	1.1 (0.8)	-0.4 to 2.6	.151	0.7 (1.0)	-1.2 to 2.7	.444	1.7 (1.0)	-0.2 to 3.6	.074†
Maternal IQ	0.07 (0.02)	0.04 to 0.1	.000**	0.04 (0.01)	0.008 to 0.06	.012*	0.03 (0.02)	-0.007 to 0.06	.110	0.05 (0.02)	0.02 to 0.09	.004**
Dose	-0.002 (0.004)	-0.009 to 0.005	.601	0.001 (0.003)	-0.005 to 0.008	.729	-0.002 (0.004)	-0.01 to 0.007	.693	0.002 (0.004)	-0.006 to 0.01	.605
VPA Dose	-0.02 (0.02)	-0.06 to 0.03	.531	-0.02 (0.02)	-0.06 to 0.03	.432	-0.04 (0.03)	-0.09 to 0.02	.184	-0.04 (0.03)	-0.09 to 0.02	.202

	Verbal Fluency			Vocabulary WBQ			Auditory Synthesis			Phoneme Deletion		
	B (SE)	95% CI	*p* Value	B (SE)	95% CI	*p* Value	B (SE)	95% CI	*p* Value	B (SE)	95% CI	*p* Value
Constant	3.3 (2.8)	-2.2 to 8.8	.241	77.6 (7.4)	63.0 to 92.1	.000	6.1 (1.8)	2.6 to 9.6	.001	1.9 (3.1)	-4.4 to 8.2	.549
CBZ	-0.7 (1.3)	-3.2 to 1.8	.577	5.7 (3.4)	-1.0 to 12.3	.094†	-0.2 (0.8)	-1.9 to 1.4	.763	1.8 (1.4)	-1.1 to 4.7	.207
LTG	1.7 (1.1)	-0.5 to 4.0	.125	5.2 (3.0)	-0.7 to 11.2	.082†	-0.1 (0.7)	-1.6 to 1.3	.866	1.7 (1.3)	-0.8 to 4.3	.183
LEV	0.6 (1.3)	-2.1 to 3.2	.671	8.1 (3.5)	1.1 to 15.1	.023*	1.2 (0.8)	-0.5 to 2.9	.175	0.9 (1.5)	-2.1 to 3.9	.545
Maternal IQ	0.06 (0.03)	0.01 to 0.1	.017*	0.3 (0.07)	0.1 to 0.4	.000**	0.01 (0.02)	-0.02 to 0.04	.523	0.07 (0.03)	0.009 to 0.1	.024*
Dose	-0.004 (0.006)	-0.02 to 0.007	.447	0.005 (0.02)	-0.03 to 0.04	.750	0.005 (0.004)	-0.002 to 0.01	.156	0.007 (0.006)	-0.006 to 0.02	.259
VPA Dose	-0.02 (0.04)	-0.1 to 0.06	.595	-0.09 (0.1)	-0.3 to 0.1	.371	-0.03 (0.03)	-0.08 to 0.02	.309	-0.1 (0.04)	-0.2 to -0.02	.017*

	Memory and Learning											
	Memory for Faces			Memory for Faces Delayed			Memory for Names			Memory for Names Delayed		
	B (SE)	95% CI	*p* Value	B (SE)	95% CI	*p* Value	B (SE)	95% CI	*p* Value	B (SE)	95% CI	*p* Value
Constant	5.1 (2.0)	1.2 to 9.0	.010	6.2 (1.9)	2.4 to 9.9	.001	5.6 (1.7)	2.1 to 9.0	.002	4.8 (2.4)	0.002 to 9.6	.050
CBZ	2.2 (0.9)	0.4 to 4.0	.015*	2.0 (0.9)	0.3 to 3.7	.022*	0.4 (0.8)	-1.2 to 2.0	.597	1.2 (1.1)	-1.0 to 3.4	.268
LTG	1.2 (0.8)	-0.4 to 2.7	.150	0.9 (0.8)	-0.6 to 2.4	.257	0.6 (0.7)	-0.8 to 2.0	.417	1.3 (1.0)	-0.6 to 3.2	.187
LEV	0.4 (0.9)	-1.4 to 2.3	.645	1.2 (0.9)	-0.6 to 3.0	.188	1.3 (0.8)	-0.3 to 3.0	.112	1.8 (1.2)	-0.5 to 4.1	.122
Maternal IQ	0.04 (0.02)	0.005 to 0.07	.024*	0.04 (0.02)	0.006 to 0.07	.022*	0.03 (0.02)	-0.004 to 0.06	.085	0.02 (0.02)	-0.02 to 0.06	.316
Dose	0.004 (0.004)	-0.004 to 0.01	.345	0.002 (0.004)	-0.005 to 0.01	.547	0.00005 (0.004)	-0.007 to 0.007	.998	-0.007 (0.005)	-0.02 to 0.003	.171
VPA Dose	-0.007 (0.03)	-0.06 to 0.05	.801	-0.05 (0.03)	-0.1 to 0.004	.069	-0.05 (0.02)	-0.1 to -0.005	.032*	-0.08 (0.03)	-0.1 to -0.008	.029*

Continued (3 of 3)

	Narrative Memory			Imitating Hand Positions			*Fine Motor Skills* Visuomotor Precision time score			Visuomotor Precision total errors		
	B (SE)	95% CI	*p* Value	B (SE)	95% CI	*p* Value	B (SE)	95% CI	*p* Value	B (SE)	95% CI	*p* Value
Constant	5.5 (1.4)	2.6 to 8.3	.000	9.7 (1.8)	6.2 to 13.3	.000	9.9 (1.7)	6.6 to 13.2	.000	3.9 (1.5)	0.9 to 6.9	.010
CBZ	0.4 (0.7)	-0.9 to 1.7	.511	0.4 (0.8)	-1.2 to 2.0	.635	-1.1 (0.8)	-2.6 to 0.4	.158	2.0 (0.7)	0.6 to 3.4	.004**
LTG	0.6 (0.6)	-0.5 to 1.8	.274	0.2 (0.7)	-1.3 to 1.6	.802	-0.9 (0.7)	-2.2 to 0.5	.194	2.3 (0.6)	1.1 to 3.5	.000**
LEV	0.6 (0.7)	-0.7 to 2.0	.363	0.6 (0.9)	-1.1 to 2.3	.491	-0.1 (0.8)	-1.7 to 1.4	.877	0.9 (0.7)	-0.5 to 2.4	.197
Maternal IQ	0.03 (0.01)	0.007 to 0.06	.014*	0.01 (0.02)	-0.02 to 0.04	.499	-0.009 (0.02)	-0.04 to 0.02	.529	0.03 (0.01)	-0.002 to 0.05	.066
Dose	-0.001 (0.003)	-0.007 to 0.005	.797	0.001 (0.004)	-0.007 to 0.008	.851	-0.001 (0.003)	-0.008 to 0.005	.670	-0.005 (0.003)	-0.01 to 0.001	.092
VPA Dose	-0.05 (0.02)	-0.09 to -0.006	.025*	0.02 (0.03)	-0.03 to 0.07	.366	-0.004 (0.02)	-0.05 to 0.04	.847	-0.003 (0.002)	-0.05 to 0.04	.877

	Fingertip Tapping repetition dominant hand			Fingertip Tapping repetition non-dominant hand			Fingertip Tapping series dominant hand			Fingertip Tapping series non-dominant hand		
	B (SE)	95% CI	*p* Value	B (SE)	95% CI	*p* Value	B (SE)	95% CI	*p* Value	B (SE)	95% CI	*p* Value
Constant	10.3 (1.1)	8.1 to 12.5	.000	12.3 (1.1)	10.2 to 14.4	.000	9.5 (1.5)	6.6 to 12.3	.000	10.3 (1.6)	7.1 to 13.5	.000
CBZ	0.5 (0.5)	-0.5 to 1.5	.317	-0.09 (0.5)	-1.0 to 0.9	.847	1.1 (0.7)	-0.2 to 2.4	.111	1.0 (0.7)	-0.5 to 2.5	.180
LTG	-0.06 (0.5)	-1.0 to 0.8	.892	-0.2 (0.4)	-1.0 to 0.6	.641	1.2 (0.6)	0.02 to 2.4	.047*	0.8 (0.7)	-0.5 to 2.1	.244
LEV	0.1 (0.5)	-1.0 to 1.2	.854	0.09 (0.5)	-0.9 to 1.1	.859	1.3 (0.7)	-0.1 to 2.6	.076†	0.9 (0.8)	-0.6 to 2.4	.254
Maternal IQ	0.02 (0.01)	-0.003 to 0.04	.102	-0.005 (0.009)	-0.02 to 0.01	.580	-0.001 (0.01)	-0.03 to 0.03	.956	-0.02 (0.01)	-0.04 to 0.01	.265
Dose	-0.002 (0.002)	-0.006 to 0.003	.516	-0.002 (0.002)	-0.006 to 0.003	.416	0.002 (0.003)	-0.004 to 0.008	.524	-0.002 (0.003)	-0.008 to 0.005	.621
VPA Dose	0.002 (0.02)	-0.03 to 0.03	.887	0.007 (0.02)	-0.02 to 0.04	.620	0.04 (0.02)	-0.003 to 0.08	.072	0.02 (0.02)	-0.03 to 0.06	.416

	Visuospatial Skills Arrows			Design Copying			Visual Attention		
	B (SE)	95% CI	*p* Value	B (SE)	95% CI	*p* Value	B (SE)	95% CI	*p* Value
Constant	4.3 (2.0)	0.3 to 8.3	.033	5.3 (1.3)	2.7 to 7.9	.000	9.9 (2.4)	5.2 to 14.6	.000
CBZ	1.6 (0.9)	-0.3 to 3.4	.093†	1.3 (0.6)	0.1 to 2.5	.033*	0.8 (1.0)	-1.3 to 3.0	.434
LTG	1.7 (0.8)	0.1 to 3.3	.036*	1.1 (0.5)	0.07 to 2.2	.037*	0.4 (1.0)	-1.5 to 2.4	.641
LEV	1.2 (1.0)	-0.7 to 3.1	.226	0.7 (0.6)	-0.6 to 1.9	.289	-0.5 (1.1)	-2.8 to 1.7	.633
Maternal IQ	0.06 (0.02)	0.03 to 0.1	.000**	0.03 (0.01)	0.005 to 0.05	.017*	0.009 (0.02)	-0.03 to 0.05	.659
Dose	-0.005 (0.004)	0.03 to 0.1	.253	-0.003 (0.003)	-0.009 to 0.002	.238	-0.001 (0.005)	-0.01 to 0.009	.856
VPA Dose	-0.01 (0.03)	-0.07 to 0.04	.644	-0.01 (0.02)	-0.05 to 0.02	.493	-0.02 (0.03)	-0.09 to 0.04	.486

e-Table 6

Multiple regression analyses with LTG as reference group

	Intelligence											
	Verbal IQ			Performance IQ			Full Scale IQ			Processing Speed Index		
	B (SE)	95% CI	*p* Value	B (SE)	95% CI	*p* Value	B (SE)	95% CI	*p* Value	B (SE)	95% CI	*p* Value
Constant	67.0 (8.3)	50.7 to 83.4	.000	78.3 (8.9)	60.7 to 96.0	.000	68.8 (8.2)	52.6 to 85.0	.000	76.5 (8.6)	59.6 to 93.5	.000
VPA	-8.5 (3.4)	-15.3 to -1.8	.014	-1.0 (3.7)	-8.4 to 6.3	.778	-5.8 (3.4)	-12.5 to 1.0	.093	-3.7 (3.6)	-10.7 to 3.4	.306
CBZ	-0.7 (3.0)	-6.7 to 5.3	.809	-2.3 (3.3)	-8.8 to 4.2	.481	-1.7 (3.0)	-7.7 to 4.2	.564	1.0 (3.2)	-5.3 to 7.2	.761
LEV	3.7 (3.3)	-2.8 to 10.2	.265	-3.0 (3.6)	-10.0 to 4.0	.402	0.5 (3.3)	-6.0 to 6.9	.882	0.9 (3.4)	-5.8 to 7.7	.789
Maternal IQ	0.4 (0.1)	0.2 to 0.6	.000	0.3 (0.1)	0.1 to 0.4	.002	0.4 (0.1)	0.2 to 0.5	.000	0.3 (0.1)	0.2 to 0.5	.000
Dose	-0.02 (0.03)	-0.1 to 0.04	.535	0.001 (0.03)	-0.1 to 0.1	.975	-0.01 (0.03)	-0.1 to 0.1	.729	-0.1 (0.03)	-0.2 to -0.04	.002
LTG Dose	0.04 (0.04)	-0.04 to 0.1	.297	-0.01 (0.04)	-0.1 to 0.1	.782	0.02 (0.04)	-0.1 to 0.1	.627	0.1 (0.04)	0.04 to 0.2	.003
	Attention and Executive functioning											
	Auditory Attention			Response Set			Statue			Design Fluency		
	B (SE)	95% CI	*p* Value	B (SE)	95% CI	*p* Value	B (SE)	95% CI	*p* Value	B (SE)	95% CI	*p* Value
Constant	4.0 (1.5)	1.0 to 6.9	.009	3.2 (1.5)	0.3 to 6.1	.032	6.7 (2.0)	2.7 to 10.6	.001	3.9 (2.3)	-0.7 to 8.6	.095
VPA	0.3 (0.6)	-0.9 to 1.5	.624	-0.7 (0.6)	-1.9 to 0.5	.224	-2.1 (0.8)	-3.7 to -0.4	.014	-2.0 (1.0)	-3.9 to -0.1	.041
CBZ	0.6 (0.5)	-0.4 to 1.7	.242	0.2 (0.5)	-0.8 to 1.3	.649	-0.3 (0.7)	-1.7 to 1.2	.708	0.3 (0.9)	-1.4 to 2.0	.741
LEV	0.3 (0.6)	-0.9 to 1.5	.643	0.1 (0.6)	-1.0 to 1.3	.802	-0.5 (0.8)	-2.0 to 1.1	.560	0.04 (0.9)	-1.8 to 1.9	.963
Maternal IQ	0.1 (0.01)	0.03 to 0.08	.000	0.1 (0.01)	0.03 to 0.08	.000	0.02 (0.02)	-0.02 to 0.06	.239	0.07 (0.02)	0.03 to 0.1	.002
Dose	-0.002 (0.006)	-0.01 to 0.009	.722	-0.01 (0.006)	-0.02 to 0.001	.075	0.004 (0.008)	-0.01 to 0.02	.602	-0.009 (0.009)	-0.03 to 0.009	.314
LTG Dose	0.001 (0.007)	-0.01 to 0.02	.832	0.01 (0.007)	-0.001 to 0.03	.060	-0.004 (0.009)	-0.02 to 0.02	.696	0.008 (0.01)	-0.01 to 0.03	.480
	Inhibition – naming total errors			Inhibition – naming time score			Inhibition- inhibition total errors			Inhibition – inhibition time score		
	B (SE)	95% CI	*p* Value	B (SE)	95% CI	*p* Value	B (SE)	95% CI	*p* Value	B (SE)	95% CI	*p* Value
Constant	7.8 (1.4)	5.0 to 10.6	.000	6.6 (1.4)	3.9 to 9.3	.000	6.9 (1.4)	4.1 to 9.7	.000	6.7 (1.2)	4.4 to 9.1	.000
VPA	-0.1 (0.6)	-1.3 to 1.1	.872	-1.4 (0.6)	-2.5 to -0.3	.015	-0.01 (0.6)	-1.2 to 1.1	.986	-0.5 (0.5)	-1.5 to 0.4	.275
CBZ	0.2 (0.5)	-0.8 to 1.2	.697	0.03 (0.5)	-1.0 to 1.0	.959	0.4 (0.5)	-0.6 to 1.4	.465	0.2 (0.4)	-0.7 to 1.1	.625
LEV	0.9 (0.6)	-0.2 to 2.0	.105	0.1 (0.5)	-1.0 o 1.2	.863	1.0 (0.6)	-0.1 to 2.1	.083	0.3 (0.5)	-0.6 to 1.3	.530
Maternal IQ	0.007 (0.01)	-0.02 to 0.03	.600	0.05 (0.01)	0.02 to 0.07	.001	0.02 (0.01)	-0.01 to 0.04	.251	0.02 (0.01)	-0.003 to 0.04	.091
Dose	-0.01 (0.005)	-0.02 to -0.002	.023	-0.009 (0.005)	-0.02 to 0.001	.092	-0.005 (0.005)	-0.02 to 0.005	.321	0.0 (0.005)	-.009 to 0.009	.923
LTG Dose	0.02 (0.007)	0.006 to 0.03	.004	0.01 (0.006)	0.001 to 0.03	.039	0.02 (0.007)	0.003 to 0.03	.019	0.006 (0.006)	-0.005 to 0.02	.294

Note. VPA: valproate; CBZ; carbamazepine; LTG: lamotrigine; LEV: levetiracetam.

Dose was standardized based on the formula: [100 x ((dose 1st trimester– median AED dose) / median AED dose)].

B = unstandardized coefficients, SE = standard error, CI = Confidence Interval.

†< .10 $^{*}p < 0.05$ $^{**}p < 0.01$

to be continued (1 of 3)

Continued (2 of 3)

						Language Skills						
	Comprehension of Instruction			Speeded Naming time score			Speeded Naming total correct			Word Generation		
	B (SE)	95% CI	*p* Value	B (SE)	95% CI	*p* Value	B (SE)	95% CI	*p* Value	B (SE)	95% CI	*p* Value
Constant	4.1	0,9 to 7.4	.013	5.4 (1.5)	2.5 to 8.3	.000	5.8 (1.9)	2.0 to 9.7	.003	5.4 (1.9)	1.6 to 9.1	.005
VPA	-2.0 (0.7)	-3.3 to -0.6	.005	-0.9 (0.6)	-2.1 to 0.3	.130	-1.5 (0.8)	-3.1 to 0.1	.066	-1.9 (0.8)	-3.4 to -0.3	.019
CBZ	-0.2 (0.6)	-1.4 to 1.0	.773	0.3 (0.5)	-0.8 to 1.4	.559	0.1 (0.7)	-1.3 to 1.5	.914	-0.5 (0.7)	-1.8 to 0.9	.511
LEV	0.6 (0.7)	-0.7 to 1.9	.373	0.2 (0.6)	-1.0 to 1.4	.726	-1.0 (0.8)	-2.5 to 0.6	.219	-0.2 (0.8)	-1.7 to 1.3	.819
Maternal IQ	0.07 (0.02)	0.04 to 0.1	.000	0.04 (0.01)	0.008 to 0.1	.010	0.03 (0.02)	-0,008 to 0.06	.127	0.05 (0.02)	0.02 to 0.1	.005
Dose	-0.008 (0.006)	-0.02 to 0.004	.211	-0.006 (0.006)	-0.02 to 0.01	.249	-0.005 (0.007)	-0.02 to 0.01	.514	-0.008 (0.007)	-0.02 to 0.006	.270
LTG Dose	0.008 (0.008)	-0.007 to 0.02	.269	0.01 (0.007)	-0.002 to 0.03	.106	0.004 (0.009)	-0.01 to 0.02	.634	0.01 (0.009)	-0.005 to 0.03	.154
	Verbal Fluency			Vocabulary WBQ			Auditory Synthesis			Phoneme Deletion		
	B (SE)	95% CI	*p* Value	B (SE)	95% CI	*p* Value	B (SE)	95% CI	*p* Value	B (SE)	95% CI	*p* Value
Constant	4.9 (2.6)	-0.3 to 10.1	.063	84.8 (7.0)	71.0 to 98.6	.000	6.3 (1.7)	2.9 to 9.6	.000	4.3 (3.2)	-2.2 to 10.7	.187
VPA	-1.6 (1.1)	-3.7 to 0.6	.154	-5.4 (2.9)	-11.2 to 0.3	.061	0.2 (0.7)	-1.2 to 1.6	.832	-0.9 (1.3)	-3.5 to 1.8	.506
CBZ	-2.3 (1.0)	-4.2 to -0.4	.017	-0.6 (2.6)	-5.6 to 4.5	.823	-0.2 (0.6)	-1.4 to 1.1	.788	0.1 (1.2)	-2.2 to 2.5	.906
LEV	-1.0 (1.0)	-3.1 to 1.0	.324	1.9 (2.8)	-3.6 to 7.4	.490	1.3 (0.7)	-0.1 to 2.6	.065	-0.7 (1.3)	-3.3 to 1.8	.578
Maternal IQ	0.1 (0.02)	0.01 to 0.1	.016	0.3 (0.1)	0.1 to 0.4	.000	0.01 (0.02)	-0.02 to 0.04	.613	0.1 (0.03)	-0.001 to 0.1	.054
Dose	-0.01 (0.01)	-0.03 to 0.01	.239	0.02 (0.03)	-0.03 to 0.1	.502	0.004 (0.01)	-0.01 to 0.02	.502	-0.001 (0.01)	-0.03 to 0.02	.960
LTG Dose	0.01 (0.010	-0.02 to 0.03	.492	-0.03 (0.03)	-0.1 to 0.03	.300	0.0 (0.01)	-0.02 to 0.02	.955	0.01 (0.02)	-0.02 to 0.04	.590
						Memory and Learning						
	Memory for Faces			Memory for Faces Delayed			Memory for Names			Memory for Names Delayed		
	B (SE)	95% CI	*p* Value	B (SE)	95% CI	*p* Value	B (SE)	95% CI	*p* Value	B (SE)	95% CI	*p* Value
Constant	6.1 (1.9)	2.5 to 9.8	.001	7.5 (1.8)	3.9 to 11.1	.000	6.1 (1.6)	2.8 to 9.3	.000	6.2 (2.3)	1.6 to 10.8	.008
VPA	-1.1 (0.8)	-2.6 to 0.4	.144	-0.6 (0.8)	-2.1 to 0.9	.410	-0.1 (0.7)	-1.4 to 1.3	.917	-0.7 (1.0)	-2.6 to 1.2	.490
CBZ	1.3 (0.7)	-0.1 to 2.6	.068	1.0 (0.7)	-0.3 to 2.4	.118	0.1 (0.6)	-1.1 to 1.3	.850	0.1 (0.9)	-1.6 to 1.8	.906
LEV	-0.5 (0.7)	-2.0 to 0.9	.486	0.3 (0.7)	-1.2 to 1.7	.703	1.1 (0.7)	-0.2 to 2.4	.105	0.7 (0.9)	-1.1 to 2.5	.439
Maternal IQ	0.04 (0.02)	0.006 to 0.1	.021	0.04 (0.02)	0.002 to 0.1	.036	0.03 (0.02)	-0.005 to 0.1	.096	0.02 (0.02)	-0.02 to 0.1	.365
Dose	-0.006 (0.007)	-0.02 to 0.008	.416	-0.001 (0.007)	-0.01 to 0.01	.882	-0.01 (0.006)	-0.03 to 0.0	.047	-0.02 (0.009)	-0.03 to 0.001	.061
LTG Dose	0.01 (0.009)	-0.004 to 0.03	.142	0.001 (0.008)	-0.02 to 0.02	.905	0.02 (0.008)	0.002 to 0.03	.030	0.01 (0.01)	-0.009 to 0.03	.275

Continued (3 of 3)

								Fine Motor Skills				
	Narrative Memory			Imitating Hand Positions			Visuomotor Precision time score			Visuomotor Precision total errors		
	B (SE)	95% CI	*p* Value	B (SE)	95% CI	*p* Value	B (SE)	95% CI	*p* Value	B (SE)	95% CI	*p* Value
Constant	6.2 (1.4)	3.4 to 8.9	.000	9.5 (1.7)	6.2 to 12.9	.000	9.1 (1.6)	6.0 to 12.1	.000	6.1 (1.4)	3.3 to 9.0	.000
VPA	-0.2 (0.6)	-1.4 (0.9)	.688	-0.3 (0.7)	-1.7 to 1.1	.639	1.0 (0.6)	-0.3 to 2.2	.142	-2.3 (0.6)	-3.5 to -1.2	.000
CBZ	-0.1 (0.5)	-1.1 to 0.9	.894	0.4 (0.6)	-0.8 to 1.6	.510	-0.2 (0.6)	-1.3 to 0.9	.711	-0.2 (0.5)	-1.3 to 0.8	.635
LEV	0.2 (0.6)	-0.9 to 1.2	.775	0.6 (0.7)	-0.7 to 2.0	.366	0.7 (0.6)	-0.5 to 2.0	.241	-1.3 (0.6)	-2.4 to -0.2	.022
Maternal IQ	0.03 (0.01)	0.005 to 0.1	.019	0.01 (0.02)	-0.02 to 0.04	.405	-0.01 (0.02)	-0.04 to 0.02	.517	0.03 (0.01)	0.0 to 0.05	.054
Dose	-0.008 (0.005)	-0.02 to 0.003	.149	-0.006 to 0.006	-0.02 to 0.01	.338	0.001 (0.01)	-0.01 to 0.01	.910	-0.01 (0.01)	-0.02 to 0.001	.080
LTG Dose	0.009 (0.006)	-0.004 to 0.02	.162	0.01 to 0.008	-0.005 to 0.03	.181	-0.002 (0.01)	-0.02 to 0.01	.757	0.01 (0.01)	-0.01 to 0.02	.481

	Fingertip Tapping repetition dominant hand			Fingertip Tapping repetition non-dominant hand			Fingertip Tapping series dominant hand			Fingertip Tapping series non-dominant hand		
	B (SE)	95% CI	*p* Value	B (SE)	95% CI	*p* Value	B (SE)	95% CI	*p* Value	B (SE)	95% CI	*p* Value
Constant	10.1 (1.1)	8.0 to 12.2	.000	12.0 (1.0)	10.1 to 14.0	.000	10.4 (1.4)	7.7 to 13.2	.000	10.8 (1.5)	7.7 to 13.8	.000
VPA	0.1 (0.4)	-0.8 to 0.9	.909	0.1 (0.4)	-0.7 to 1.0	.748	-1.5 (0.6)	-2.6 to -0.4	.011	-0.9 (0.6)	-2.2 to 0.3	.153
CBZ	0.7 (0.4)	-0.1 to 1.5	.082	0.1 (0.4)	-0.7 to 0.8	.792	-0.1 (0.5)	-1.1 to 0.9	.869	0.3 (0.6)	-0.8 to 1.4	.559
LEV	0.3 (0.4)	-0.06 to 1.1	.525	0.3 (0.4)	-0.5 to 1.1	.491	0.1 (0.6)	-1.0 to 1.2	.891	0.2 (0.6)	-1.0 to 1.4	.726
Maternal IQ	0.02 (0.01)	-0.002 to 0.04	.081	-0.01 (0.01)	-0.2 to 0.01	.616	0.001 (0.01)	-0.02 to 0.03	.928	-0.01 (0.01)	-0.04 to 0.01	.321
Dose	-0.006 (0.004)	-0.01 to 0.002	.132	-0.001 (0.004)	-0.01 to 0.01	.728	0.002 (0.01)	-0.01 to 0.01	.659	-0.01 (0.01)	-0.02 to 0.01	.418
LTG Dose	0.006 (0.005)	-0.004 to 0.02	.221	-0.001 (0.01)	-0.01 to 0.01	.861	0.001 (0.01)	-0.01 to 0.01	.925	0.01 (0.01)	-0.01 to 0.02	.496

			Visuospatial Skills						
	Arrows			Design Copying			Visual Attention		
	B (SE)	95% CI	*p* Value	B (SE)	95% CI	*p* Value	B (SE)	95% CI	*p* Value
Constant	6.3 (1.9)	2.5 to 10.0	.001	6.3 (1.2)	3.9 to 8.8	.000	10.3 (2.2)	5.8 to 14.7	.000
VPA	-1.7 (0.8)	-3.3 to -0.2	.029	-1.0 (0.5)	-2.0 to 0.01	.052	-0.1 (0.9)	-2.0 to 1.7	.879
CBZ	-0.3 (0.7)	-1.7 to 1.0	.631	0.3 (0.5)	-0.6 to 1.2	.542	0.5 (0.8)	-1.1 to 2.2	.515
LEV	-0.7 (0.8)	-2.2 to 0.8	.342	-0.3 (0.5)	-1.3 to 0.6	.496	-0.8 (0.9)	-2.6 to 0.9	.350
Maternal IQ	0.1 (0.02)	0.03 to 0.1	.001	0.03 (0.01)	0.01 to 0.1	.015	0.01 (0.02)	-0.03 to 0.1	.669
Dose	-0.001 (0.01)	-0.02 to 0.01	.857	-0.01 (0.01)	-0.02 to 0.002	.113	-0.01 (0.01)	-0.02 to 0.01	.421
LTG Dose	-0.01 to 0.01	-0.03 to 0.01	.419	0.01 (0.01)	-0.01 to 0.02	.295	0.01 (0.01)	-0.01 to 0.03	.468
